# Nursing’s Potential to Address the Growing Cancer Burden in Low- and Middle-Income Countries

**DOI:** 10.1200/JGO.2015.001974

**Published:** 2016-02-03

**Authors:** Julia M. Challinor, Annette L. Galassi, Majeda A. Al-Ruzzieh, Jean Bosco Bigirimana, Lori Buswell, Winnie K.W. So, Allison Burg Steinberg, Makeda Williams

**Affiliations:** **Julia M. Challinor,** International Network for Cancer Treatment and Research, Brussels, Belgium; **Annette L. Galassi and Makeda Williams,** National Cancer Institute, Bethesda, MD; **Majeda A. Al-Ruzzieh,** King Hussein Cancer Center, Amman, Jordan; **Jean Bosco Bigirimana,** Inshuti Mu Buzima, Rwinkwavu, Rwanda; **Lori Buswell,** Partners in Health, Boston, MA; **Winnie K. W. So,** The Chinese University of Hong Kong, Hong Kong, Special Administrative Region, People’s Republic of China; and **Allison Burg Steinberg,** Johns Hopkins Sidney Kimmel Comprehensive Cancer Center at Sibley Memorial Hospital, Washington, DC.

## INTRODUCTION

Cancer has become a major noncommunicable disease (NCD) in low- and middle-income countries (LMICs) where an inadequately prepared and insufficient nursing workforce exists. Successful cancer care requires a team approach, and knowledgeable oncology nurses play a crucial role in a functioning team. The goals of reducing cancer incidence, improving survival, and providing better palliative care cannot be met without the efforts of these nurses. Oncology nurses who work in the community and at the bedside can deliver needed patient, family, and community education; implement early detection programs; administer treatments; identify complications; provide palliative care; and lead and collaborate on clinical research. Well-prepared oncology nurses have demonstrated a wide-ranging impact across the spectrum of cancer care in high-income countries (HICs). To benefit from this expertise, LMICs need workforce capacity–building efforts to educate nurses in cancer care initiatives, expand the scope of nursing practice, and increase task sharing as demonstrated in HIV/AIDS care in LMICs^1^; these efforts require time, money, and political commitment.

Despite efforts by western partners that work in LMIC oncology settings to include nursing training and an expansion of practice in their programs, results are limited and rarely, if ever, evaluated to determine efficacy.^2,3^ Therefore, all stakeholders involved in cancer control, treatment, education, and research (ie, ministries of health and education, nongovernmental organizations, funders, cancer advocates) must include a scale-up of oncology nursing in LMIC national and regional cancer control planning.

## LMIC NURSES AND THE CANCER CONTINUUM

This article builds on these efforts and focuses on the critical role that nurses with appropriate education can play in providing care to patients with cancer across the cancer continuum to help to improve cancer control in LMICs and create an additional point of access to cancer care, particularly in settings where specialized care is rare and existing resources are strained. By highlighting the ways nurses can contribute to the improvement of oncology care in LMICs, this report addresses the issues that face the workforce and includes recommendations to illustrate how health and educational systems can be used to strengthen the expertise and expand the role of oncology nurses in LMICs. Nursing education, the nursing practice environment, and opportunities for role expansion and research have not kept pace with the growing need.^4^ The intention of this article is to serve as a call to action to focus attention and resources on the need for education and training of oncology nurses in LMICs.

## NURSING’S POTENTIAL CONTRIBUTIONS TO CANCER CARE: WHAT CAN BE DONE IN LMICS

In all aspects of the fight against cancer, nurses participate dynamically as part of an interdisciplinary team. A well-prepared oncology nursing workforce includes generalist nurses who are prepared at the basic level and provide health promotion, risk assessment, and care for people who receive cancer treatment in their general practice; specialized nurses whose primary focus is the delivery of cancer care and who care mostly for people with or at risk for cancer; and advanced practice oncology nurses who provide cancer care at the master’s degree level of education or higher.^5^ Oncology nurses with master’s and doctoral education contribute through advanced practice, education, and scientist roles.

Adequately educated nurses can play many vital roles across the cancer control continuum; however, not all countries use nurses to their fullest capacity. As part of their Global Action Plan for NCDs, the WHO recommended that nations “[optimize] the scope of nurses’ and allied health professionals’ practice to contribute to the prevention and control of non-communicable disease, including addressing barriers to that contribution,”^6p42^ and many opportunities exist to maximize the impact of oncology-trained nurses in addressing the cancer burden.

Nurses’ intimate knowledge of patient populations make them an obvious partner among oncology specialists to address the increasing public health burden of this group of diseases. Nurses trained as specialists in oncology could address public health cancer risks, such as smoking and obesity, as well as cancer-causing environmental and occupational hazards in their local areas. Oncology nurse researchers in LMICs could collaborate with epidemiologists, medical anthropologists, environmental health scientists, public health professionals, and health economists to gather the much-needed data to measure the efficacy of cancer prevention activities, cancer incidence and prevalence, and outcomes of people treated for cancer. This section outlines what has been done in oncology from prevention through end-of-life care and survivorship by nurses who work in countries of various resource levels and what can be done in LMICs specifically to improve cancer-related outcomes around the globe.

### Cancer Education

In LMICs, a patient’s first encounter with the health system often is with a community health worker (CHW) at the dispensary (community) level; however, CHW and nurses’ knowledge of cancer and cancer risk factors, signs, and symptoms in many LMICs is low.^7-10^ Oncology nurses placed throughout the country could dramatically increase the number of cases diagnosed in early stages. In addition, oncology nurses can educate CHWs about cancer to raise awareness and appropriately refer a patient for further evaluation.^7,11^ Further, nurses can increase adherence to screening guidelines because they are viewed as trusted members of their society.^6^ Thus, nurses can act more efficiently as patient navigators through the continuum of care to link the patient to local health systems and decrease delay in care.^12,13^

### Prevention: The Unique Role of Nurses

One-third or more cancer diagnoses can be avoided by reducing risk factors, such as tobacco use, poor diet, low levels of physical activity, alcohol consumption, workplace and environmental carcinogens, and exposure to radiation; immunizing against hepatitis B and C viruses and the human papillomavirus (HPV); and preventing infection with *Helicobacter pylori* and schistosomiasis.^14^ Nurses are uniquely positioned to successfully implement preventive interventions at both the individual and the community level given their accessibility to and active role in the community. Disease prevention and health promotion have long been a part of nursing’s scope of practice. Nurses are trained to identify risk factors, and they have the communication and teaching skills to work with individuals, families, and communities to change behaviors to reduce risk factors.^15-17^

A Cochrane review of 16 studies about tobacco cessation conducted in various HICs showed evidence that the likelihood of reaching the positive outcome of quitting smoking is increased when nursing-specific interventions are used.^18^ Emerging research has demonstrated that nurses are instrumental in providing effective smoking cessation intervention in LMICs as well.^19^ In vaccination programs, nurses also have been instrumental in educating the public about the importance of vaccinations and in implementing these programs in LMICs for viruses such as HPV, the cause of cervical cancer, and hepatitis B virus, which is associated with an increased incidence of liver cancer. A school-based, opt-out HPV vaccination program in Rwanda has used nurses to reduce the two-decade delay in vaccine introduction between HICs and LMICs to 5 years.^20^

### Screening and Early Detection: Opportunities for Expanded Care

To improve outcomes, prevention must be coupled with screening and early detection measures. Early detection decreases the overall costs of cancer treatment.^9,21^ With additional training, nurses can perform the broad range of interventions that contribute to screening,^17,22^ early detection, and even treatment of precancerous lesions.^6^ With nurses performing these activities, the few available physicians and oncologists can focus on tasks that require their specialized skills.^23,24^

Various programs have demonstrated that nurses play a vital role in screening. Studies in HICs have shown that nurses can perform flexible sigmoidoscopy or colonoscopy with ratings in patient satisfaction, safety, and effectiveness similar to those for procedures done by general surgeons and GI fellows.^25-28^ These findings suggest that appropriately trained oncology nurses in LMICs could assume this level of care as part of the task shifting that has already been demonstrated to be successful (eg, for colposcopy). However, challenges related to scope of practice for oncology nurses in LMICs remain.

Research findings in the Philippines, Indonesia, and Malaysia have demonstrated that clinical breast examinations done by nurses are a sustainable form of early detection and primary screening.^29-31^ In promoting general breast health awareness, nurses are a well-suited professional group to destigmatize disease within their respective communities.^11^ Nurses in LMICs knowledgeable about breast health and with skills in clinical breast examination can contribute to reaching the goal of downstaging the presentation of breast cancer in these countries.^32^

The Cervical Cancer Prevention Program in Zambia demonstrates the value nurses provide in cervical cancer control through the Screen and Treat program.^33^ Similar programs have been successfully implemented in other LMICs, including India.^34^

### Treatment: Roles in Surgery, Chemotherapy, and Radiotherapy

Nurses around the globe play a vital and central role in the delivery of all cancer treatment modalities, particularly surgical, radiation, and medical oncology.^35^ For patients undergoing surgical intervention, nurses teach patients what to expect before, during, and after procedures. The nurse assesses the patient during the postoperative period by monitoring wound healing, preventing infection, managing pain, and facilitating return to activities of daily living. Surgical interventions such as mastectomy, orchiectomy, or colectomy may require nurses to assist patients to adapt to altered physical or emotional functioning. Surgical nurses in LMICs participate in multidisciplinary training to improve surgical outcomes in people with cancer.^36^ Despite this evidence, nursing management and care of patients undergoing surgical intervention remain suboptimal in many countries due to lack of adequate resources and targeted nursing education. For example, in Paraguay, postoperative pediatric neurosurgical management does not prevent the high infection rate that leads to early mortality for children with brain tumors.^37^ Well-educated pediatric oncology nurses could support efforts to improve neurosurgical interventions in LMICs.

Although radiation therapy is an important component of cancer control,^38^ of the 139 LMICs (as categorized by the World Bank), almost one-half (55) have no radiation facilities, and only four have adequate equipment.^39^ Nurses who work in radiation oncology units clinically assess and educate patients about radiotherapy by addressing patient fears and providing information about potential adverse effects. Nurses need to be knowledgeable about the specific radiation field so that they can educate patients to identify adverse effects early and so that steps can be taken to avoid complications, avoid treatment delays, or conclude radiation treatment prematurely, as has been demonstrated in Indonesia.^40^

Nurses who work in medical oncology practices in LMICs often administer and prepare chemotherapy^41,42^ and generally without personal protective equipment or a biologic safety cabinet.^43,44^ They need to be meticulous in this practice, particularly in math calculations to reconstitute chemotherapy agents, calculate body surface area, and assure appropriate dosing that is consistent with treatment protocols. Many nurses in LMICs, however, are not formally trained in chemotherapy administration and would like further education on the subject.^45^

Because of the lack of education, protective equipment, and trained pharmacists in many locations around the globe, chemotherapy preparation and administration poses a significant risk to nurses. Training for the safe handling of hazardous drugs is essential to protect both the nurses and their patients and families.^46^ Personal protective equipment, which includes chemotested gloves, disposable gowns, masks with eye shields or goggles, and a biosafety cabinet, must be available and used properly to protect nurses within their practice setting.^43,47,48^

Chemotherapy preparation ideally is the scope of the pharmacist and should be conducted in appropriate pharmacy facilities, even in LMICs.^38^ In fact, the inclusion of an oncology-trained pharmacist is a cost-effective way for an LMIC setting to improve chemotherapy preparation and handling; nurse and patient safety; and inventory control, patient and family education, hazardous drug waste management, and pharmaceutical cost savings.^42^ Nonetheless, the reality in many LMICs is that a shortage of either educated pharmacists or safety equipment exists and that nurses ultimately are responsible for both preparing and administering chemotherapy and therefore need to be properly educated about safety measures. Other professionals, such as engineers, are needed to develop low-cost, effective chemotherapy preparation facilities and equipment.

### Treatment: Provision of Supportive Care in All Modalities

Patients who undergo cancer treatment benefit from supportive care, or the prevention and management of adverse effects.^49-51^ Oncology nurses are skilled at conducting a comprehensive assessment of the health and supportive care needs of patients with cancer. In addition, oncology nurses can educate and provide psychosocial and spiritual support by sharing and applying their knowledge of cancer and treatment modalities and adverse effects.^5,52^

Cancer and treatment-related symptoms are major stressors in patients with cancer. In LMICs, symptom burden that patients with cancer experience may be even worse than the experience of patients in HICs because supportive care, including symptom management, often is a low priority in LMICs, and essential medicines (eg, to control pain) may be unavailable.^53,54^ However, lack of information and education about cancer treatment and the management of its adverse effects are major barriers to effective cancer treatment in LMICs because supportive care often is inadequately addressed.^32,55,56^

Various studies have reported that patients who do not receive adequate symptom management can experience an increase in psychological distress, treatment delays or noncompliance, a prolonged hospital stay, and negative effects on quality of life.^52,57-59^ By educating patients so that they have a better understanding about their treatment, adherence to treatment could be enhanced and, in turn, could result in better treatment outcomes.^60-62^

Studies in HICs have reported positive effects of nursing interventions on the management of symptoms^57,63,64^ to minimize the consequence of cancer treatment^57,65,66^ and promote quality of life^67-69^ among patients. Oncology nurses are the closest to the patient and, therefore, have the unique opportunity and privilege to advocate for and support the patient and his or her family. They also play a significant role in assuring that the highest quality of care is delivered to achieve the best possible outcome.^70,71^

### An Opportunity to Improve Outcomes Through Research and Evidence-Based Practice

Nurses have a unique perspective on health care systems that can contribute effectively to research and evidence-based interventions that inform service delivery, education and training, and policy recommendations. Nursing research has had a significant impact on health promotion and disease prevention in LMICs for health issues such as HIV/AIDS,^72-74^ maternal and child health,^75,76^ and mental health^77^ and can have a similar impact on oncology care. Nursing research and multidisciplinary research led by a team of nurses, physicians, statisticians, and others could assist in developing strategies for resource-appropriate best practices. In addition, nurses could make pivotal contributions to translational research for the development of evidence-based practice.

The WHO Global Forum for Government Nursing and Midwifery Officers (the Global Forum) calls for nursing and midwifery research on efficacy and cost-effectiveness of interventions, translational research, and collaborative partnerships for funding research and innovative projects.^19^ To enhance nurse and midwife capacity to address NCDs such as cancer, the Global Forum suggests research focused on expanding settings for implementing interventions, integrating risk assessment with clinical practice, surveying the prevalence of risky health behaviors, and training to enhance knowledge and skills on cancer and its risk factors.^19^ The advancement of nursing research in LMICs not only will enhance the cadre of cancer researchers^78^ but also will improve service delivery, training, policy, and health outcomes.^79,80^

As a result of challenges that stem from the hierarchy of power; lack of resources, mentors, and subject matter experts; limited oncology education and training opportunities; and lack of funding, nurses in LMICs interested in research usually have fewer opportunities compared with colleagues in HICs.^81^ They must also confront the perception that nurses are not fit to be researchers and the lack of integration of research with service delivery, treatment, and care. Capacity building for nurse-led research in LMICs is critical in elevating nurses as research scholars,^82^ translating nursing research into evidence-based practice, and recognizing nurses as potential policymakers,^83^ which thus improves health outcomes.

### Palliative Care and Hospice: Managing Symptoms From Diagnosis Through End of Life

Palliative care supports patients with cancer and their families throughout the trajectory of illness. It might become even more significant in countries where late-stage diagnosis, for example,^84^ and low resource levels for treatment are most prevalent.

As with care during treatment, nurses assess, identify, and manage not only pain but also the physical, psychosocial, spiritual, and cultural needs of patients and their families at the end of life.^85^ Nurses trained in pain management; palliative care; and management of grief, death, and dying can positively affect the end-of-life experience, help patients to achieve a peaceful death, and help their families to cope with loss and grief.^86-88^ These skills are especially important for nurses in LMICs where the majority of patients present with advanced disease and where effective treatments to cure or even control the disease are limited.^44,89^

It is estimated that of the 20 million people needing palliative care at the end of life, around 80% live in LMICs.^90^ In most LMICs, however, palliative care is not considered an essential part of cancer care,^91^ and the majority of these countries do not meet basic international guidelines for the delivery of palliative care.^90,92^ Barriers to effective palliative care in LMICs include the limited availability of opioids and other medications to manage symptoms, inadequate knowledge, and a lack of country-level palliative care policies or integrated services.^89,93,94^ The WHO estimates that 5.5 million patients with cancer die in pain annually because they do not have access to opioid medications.^95^ Reasons include legal and regulatory restrictions on the use of opioids due to concerns about diversion, addiction, and misuse and cultural perceptions about pain and its treatment.^96^

Efforts are under way to improve nurses’ ability to provide adequate end-of-life care, such as the End-of-Life Nursing Education Consortium curriculum, which has been adapted to serve the needs of an international nursing audience. This curriculum has been used to train nurses from LMICs, including Eastern Europe, Central Asia, and Africa.^97^ The Institute of Hospice and Palliative Care in Uganda also leads regional efforts to train nurses.^98^

Nurses can act as advocates for improved end-of-life care in their country. They can work with government officials and nongovernmental organizations to develop policies that improve the availability of opioids. When nurses lead and participate in the development of hospice and palliative care services, access is expanded.^96,99^ Because nurses often are the health care provider with the closest connection with the community, they can help to overcome cultural barriers against the use of opioid medications by educating patients, families, and their colleagues.^100^

### Survivorship

The term cancer survivor, as used in this section, is defined as individuals who have completed primary cancer treatment.^101^ The incidence of cancer has been increasing steadily in the past decade as has the number of cancer survivors. In 2012, 14.1 million people were given a diagnosis of cancer, and globally, 32.6 million people have lived within 5 years of diagnosis^102,102a^; however, the prolonging of the lifespan of survivors does not necessarily mean that their well-being is improved. Cancer survivors continue to experience late effects of treatment and psychosocial complications after treatment.^103,104^ Cancer survivors have been reported to experience a poorer quality of life than the general population.^105,106^ Prolonged experience of complications and a poorer quality of life may be detrimental to survivors’ health and, in turn, increase health care use and the burden on existing health care services.^107,108^ Although health care professionals in HICs have highlighted the use of cancer survivorship care plans,^109^ which aim to assist in providing a patient with a smooth transition from active treatment at a cancer center to post-treatment care in the community,^110^ this kind of supportive care service for cancer survivors in LMICs has not been addressed.

Various studies have reported positive outcomes of health care services for survivors that are led by nurses or include nurse involvement. Nurse-led efforts in psychosocial support and healthy lifestyle promotion have shown benefits for quality of life and behavioral outcomes for patients,^111,112^ and nurses have been involved in protocol design for post-treatment follow-up care.^113^ Although most of the studies were conducted in HICs, the findings provide strong evidence that nurses in LMICs who received oncology-specific education and training could make comparable contributions to supportive care services for cancer survivors.

Across the cancer continuum and around the world, nurses play an integral role throughout a patient’s cancer journey. Nurses must be educated and positioned to practice to their utmost potential to improve patient outcomes at all points of care and in all locations.^114^

In conclusion, the burden of cancer is increasing worldwide,^115^ and cancer is one of the NCDs prioritized by the agendas of the WHO, the United Nations, and other international organizations. Unfortunately, the health care professionals who provide care across the cancer continuum, from prevention and detection to treatment, end-of-life care, and survivorship, are seriously lacking in LMICs. Initiatives have addressed cancer in LMICs by using the limited health care staff available; however, the current mortality statistic for patients with cancer in these countries is 72% to 75%, which is devastating.^116^ When governments and donors commit to supporting sustainable development of the health care infrastructure, including the personnel and material resources required, this statistic can be changed, and nurses must be central to designing the solutions.
